# Dr. Havelock Ellis

**Published:** 1899-01

**Authors:** 


### DR. HAVELOCK ELLIS.

We learn with regret, which will, we feel, be shared by all the scientific world, that this gited author has decided not to publish, in England, his great work on "Sexual Inversion," or the completion of his series of announced volumes on the "Psychology of the Sex," of which only the initial volume has appeared.

This action has been due to the conduct of his publisher, Mr. George Bedborough, who was indicted for the selling of the work as "wicked, lewd, impure, scandalous and obscene," and who, although Mr. Ellis had retained eminent counsel and raised a considerable sum of money to provide for the defense, shortly before the trial came on, last October, without consulting those whose support he had accepted, plead guilty to a part of the charge, and Mr. Ellis and his counsel were unable to obtain even a hearing upon the trial; and the defendant, in pursuance of an arrangement made with the prosecuting counsel and the Recorder (Sir Charles Hall), was discharged upon his own recognizancc.

It is incredible that such scandalous conduct, on the part of the authorities and the Recorder, should pass unnoticed and unrebuked.

The animus of the Recorder might be in doubt, if it were not for his language on the occasion. He said from the bench to the prisoner: "You might at the outset, perhaps, have been gulled into the belief that somebody might say this was a scientific book. But it is impossible for anybody with a head on his shoulders, to open the book without seeing that it is a pretense and a sham, and that it is merely entered into for the purpose of selling this filthy publication."

This officer has never been credited with being a person of any scientific attainments. That his education upon such questions has been seriously neglected is established by his own testimony.

It is doubtful if any scientific authority on the continents of Europe or America, or in the British Islands, would agree with the language he used. He has, by an illegal and reprehensible abuse of his position, consented to the substantial release of the publisher, without punishment, whom he believed was guilty of a crime, and stabbed in the back, and without anv excuse or opportunity of being heard, one of the most brilliant of the British writers, of his time and our era, on subjects connected with anthropology; and no matter what his past career or character may have been, he will receive, as he justly deserves, the contempt of the whole scientific world.

The honorable career of Mr. Havelock Ellis cannot be seriously compromised by such an unmerited assault from the bench, and it is a marvel how our English cousins, with their love of fair play, would submit to such aspersions upon the motives of a gentleman of Mr. Ellis' character.

There can be no doubt that Mr. Ellis made a serious mistake in selecting his publisher. Mr. Bedborough may have been a dealer in obscene books, and *persona non grata* with the Court or the authorities. It is stated that he has published a magazine devoted to the propaganda of unconventional views upon marriage, and the book of Mr. Ellis may have been, in the judgment of the authorities and the Recorder, tarred with the same stick with Mr. Bedborough's other publications—we do not know. Even were this so (which was, as we think, a grave error on the part of Mr. Ellis), it would not change the character of the unjust accusation made against the work by the Recorder, but would bear in mitigation and explanation of the judicial utterances; but the lesson to Mr. Ellis, and to writers generally, would remain, that authors, as well as men, are known and judged by the company they keep, and Mr. Ellis may, like poor Tray, have been cudgelled by reason of his unfortunate environment.—*Medico-Legal Journal.*

